# An EM algorithm based on an internal list for estimating haplotype distributions of rare variants from pooled genotype data

**DOI:** 10.1186/1471-2156-14-82

**Published:** 2013-09-13

**Authors:** Anthony YC Kuk, Xiang Li, Jinfeng Xu

**Affiliations:** 1 Department of Statistics and Applied Probability, National University of Singapore, Singapore, 117546, Singapore; 2 Division of Biostatistics, Department of Population Health, New York University School of Medicine, New York, NY 10016, USA

**Keywords:** Collapsed data, EM algorithm, Haplotype frequency estimation, Haplotype list, Pooled genotype data, Rare variants

## Abstract

**Background:**

Pooling is a cost effective way to collect data for genetic association studies, particularly for rare genetic variants. It is of interest to estimate the haplotype frequencies, which contain more information than single locus statistics. By viewing the pooled genotype data as incomplete data, the expectation-maximization (EM) algorithm is the natural algorithm to use, but it is computationally intensive. A recent proposal to reduce the computational burden is to make use of database information to form a list of frequently occurring haplotypes, and to restrict the haplotypes to come from this list only in implementing the EM algorithm. There is, however, the danger of using an incorrect list, and there may not be enough database information to form a list externally in some applications.

**Results:**

We investigate the possibility of creating an internal list from the data at hand. One way to form such a list is to collapse the observed total minor allele frequencies to “zero” or “at least one”, which is shown to have the desirable effect of amplifying the haplotype frequencies. To improve coverage, we propose ways to add and remove haplotypes from the list, and a benchmarking method to determine the frequency threshold for removing haplotypes. Simulation results show that the EM estimates based on a suitably augmented and trimmed collapsed data list (ATCDL) perform satisfactorily. In two scenarios involving 25 and 32 loci respectively, the EM-ATCDL estimates outperform the EM estimates based on other lists as well as the collapsed data maximum likelihood estimates.

**Conclusions:**

The proposed augmented and trimmed CD list is a useful list for the EM algorithm to base upon in estimating the haplotype distributions of rare variants. It can handle more markers and larger pool size than existing methods, and the resulting EM-ATCDL estimates are more efficient than the EM estimates based on other lists.

## Background

In statistical genetics, the haplotype distribution is the joint distribution of the allele types at, say, *L* loci. We will focus on bi-allelic loci in this article so that each haplotype vector is a vector of binary values, and the haplotype distribution is a multivariate binary distribution. The importance of haplotypes is well documented [1-3] and reinforced more recently by the works of Muers [4] and Tewhey *et al.*[5]. By incorporating linkage disequilibrium information from multiple loci, haplotype-based inference can lead to more powerful tests of genetic association than single-locus analyses. Haplotype distributions are usually estimated from individual genotype data which is the sum of the maternal and paternal haplotype vectors of an individual. As reviewed by Niu [6] and Marchini *et al.*[7], statistical approaches to haplotype inference based on individual genotype data are effective and cost-efficient. These include the expectation-maximization (EM) type algorithms for finding maximum likelihood estimates (MLE) [8], and the Bayesian PHASE algorithm [9]. Since DNA pooling is a popular and cost-effective way of collecting data in genetic association studies [10-14], the EM algorithm and its variants have been extended by various authors [15-18] to handle pooled genotype data (i.e., the sum of all 2*k* haplotype vectors of all *k* individuals in a pool), whereas Pirinen *et al.*[19], Gasbarra *et al.*[20] and Pirinen [21] have extended Bayesian algorithms using Markov Chain Monte Carlo (MCMC) or reversible jump MCMC schemes. Also from a Bayesian perspective, Iliadis *et al.*[22] conduct deterministic tree-based sampling instead of MCMC sampling, but their algorithm is feasible for small pool sizes only, even though the block size can be arbitrary. Despite the falling costs of genotyping, the popularity of the pooling strategy has not waned, with Kim *et al.*[23] and Liang *et al.*[24] advocating the use of pooling for next-generation sequencing data. The importance of pooling increases with the recent surge of interest in rare variant analysis based on re-sequencing data [25] to explain missing heritability [26] and diseases that cannot be explained by common variants. Roach *et al.*[27] predict that “haplotypes that include rare alleles … will play an increasingly important role in understanding biology, health, and disease”. Perhaps more so than in the analysis of common variants, pooling has an important role to play in the analysis of rare variants. This is because the standard methods for testing genetic association are underpowered for rare variants due to insufficient sample size as only a small percentage of study subjects would carry a rare mutation, and pooling is a way to increase the chance of observing a rare mutation. By using a pooling design, we could include more individuals in a study at the same genotyping cost. The study by Kuk *et al.*[28] shows that pooling does not lead to much loss of estimation efficiency relative to no pooling when the alleles are rare.

Our focus is on developing computationally feasible EM-type algorithms to estimate haplotype frequencies of rare variants from pooled genotype data. There are two main impediments to the use of EM algorithm in estimating haplotype distribution from pooled genotype data. First, the number of putative haplotypes grows exponentially with the number of loci. Secondly, things get worse when pool size increases as the number of individual haplotype configurations compatible with the observed pool totals quickly becomes astronomical. As a result, the EM algorithm can only be applied to data with small to moderate number of loci and pool size. For example, Gasbarra *et al.*[20] commented that without prior knowledge or restriction on the possible haplotypes, existing algorithms cannot handle the case of 21 loci with pool size 6. We have recorded running times of 1862 and 2900 seconds on an intel (R) Core (TM) desktop when the traditional EM algorithm is applied to pooled genotype data with 12 loci for 74/37 pools of size 2/4 each. Gasbarra *et al.*[20] advocate the use of database information to create a list of frequently occurring haplotypes. By combining this idea of using database information to create a list with a normal approximation [17] for the density of the pooled allele frequencies, Pirinen [21] proposed an AEML (Approximate EM with List) algorithm which runs much faster than the unrestricted EM algorithm.

We do not assume the existence of an external list for two reasons. First, database information for rare alleles is currently still lacking. Secondly, an EM type algorithm restricted to a list is sensitive to the correct choice and completeness of the external list used. Instead, we use the data on hand to construct an internal list. Motivated by the collapsed data estimation method developed by Kuk *et al.*[29] which only keeps track of whether an allele count is “0” or “ ≥1”, we propose a collapsed data (CD) list of possible haplotypes. It will be shown in the Methods section that for rare genetic variants, the CD list has inflated probabilities of capturing the true underlying haplotypes. To improve coverage, we augment the CD list by adding those haplotypes with only one “1” (i.e., only one rare variant occurs) to result in an augmented CD (ACD) list. The EM algorithm restricted to the ACD list still does not perform satisfactorily in our simulation studies, apparently due to the inclusion of too many false haplotypes. In response, we propose an ATCD (augmented and trimmed CD) list where those haplotypes with estimated frequencies lower than a threshold at each iteration of the algorithm are removed from the list. We propose a method to select the threshold by benchmarking the resulting EM estimate of the frequency of the ancestral haplotype of all zeros (i.e., no variant occurs) with the corresponding estimate obtained using the collapsed data method of Kuk *et al.*[29]. To assess the performance of the various estimators, we simulate genotype data resembling those collected for the 148 obese individuals in the CRESCENDO cohort study http://clinicaltrials.gov/ct/show/NCT00263042, at 25 loci near the *MGLL* gene on chromosome 3, and 32 loci near the *FAAH* gene on chromosome 1. The EM estimates based on the CD list and the ACD list do not perform well in the simulation study. In particular, they over-estimate the haplotype frequency of the ancestral haplotype of all zeros. The EM estimates based on the ATCD list, on the other hand, perform very well. In the two scenarios involving 25 and 32 loci, the EM-ATCDL estimates outperform the EM estimates based on other lists as well as the collapsed data maximum likelihood estimates. We conclude that the augmented and trimmed CD list is a useful list for the EM algorithm to base upon in estimating the haplotype distributions of rare variants.

## Results

To identify rare genetic variants associated with obesity, investigators of the CRESCENDO cohort study obtained re-sequenced data for 148 obese persons and 150 controls around two genes known to be involved in endocannabinoid metabolism: *FAAH* on chromosome 1, and *MGLL* on chromosome 3. There are 31Kbp of re-sequenced data near the *FAAH* gene, and 157Kbp near the *MGLL* region. Bhatia *et al.*[30] discovered two 5Kbp regions enriched in rare variants (RVs) located just upstream of the *FAAH* and *MGLL* genes respectively, with 32 RVs in the first region, and 25 RVs in the second region. To estimate the underlying haplotype distributions, we apply the algorithms proposed in this paper, as well as the EM with a list (EML) method described in Kuk *et al.*[29], where the list is determined combinatorially. The collapsed data maximum likelihood estimates (CDMLE) are also computed. To save space, we only report the estimates based on the obese individuals, which is the more interesting case, as there are very few mutations among the control subjects. Table 1 reports the CDMLE’s, as well as the estimates obtained using EML, EM-CDL (EM with CD list) and EM-ATCDL (EM with augmented and trimmed CD list) algorithms for the 25 loci case. The estimates on the left panel (*k*=1) are based on individual genotype data, whereas the right panel (*k*=2) estimates are based on pooled genotype data that result from grouping the 148 obese individuals into 74 pools of size 2 each. Obviously, the estimates based on 148 pools of size 1 (i.e., individual genotype data) should be more reliable than those based on 74 pools of size 2, and so we should use the estimates on the left panel of Table 1 as the benchmark. It is interesting to note that as the pool size *k* increases to 2, the CDMLE, EML and EM-CDL estimates remove some haplotypes that are assigned probabilities in the *k*=1 case, and in their place, some other haplotypes not presented in the *k*=1 case are assigned probabilities in the *k*=2 case. We will see later in the Methods section that it is an inherent property of the CD list to include extraneous false haplotypes as pool size increases. By augmenting and trimming the CD list in the proposed way, the EM-ATCDL estimates based on *k*=1 and 2 are much more comparable with similar support, which is desirable.

**Table 1:** **Haplotype frequency estimates in the *MGLL * region using data from 148 obese individuals**

	***k=1, n=148***				***k=2, n=74***				
**Position of ‘1’s**	**CDMLE**	**EML**	**EM-CDL**	**EM-ATCDL**		**CDMLE**	**EML**	**EM-CDL**	**EM-ATCDL**
None	0.7927	0.7941	0.7995	0.7984		0.7912	0.8202	0.8169	0.7898
1	0.0536	0.0505	0.0509	0.0544		0.0497	0.0397	0.0398	0.0494
2	0.0043	0.0034	0.0034	0.0034					0.0034
3	0.0456	0.0433	0.0436	0.0441		0.0381	0.0291	0.0291	0.0440
5	0.0043	0.0034	0.0034	0.0034					0.0034
6	0.0043	0.0034	0.0034	0.0034		0.0067	0.0034	0.0034	0.0034
9	0.0085	0.0072	0.0073	0.0103		0.0133	0.0079	0.0079	0.0101
11	0.0043	0.0034	0.0034	0.0034					0.0034
14									0.0034
15	0.0043	0.0034	0.0034	0.0034					0.0034
19	0.0043	0.0068	0.0068	0.0068		0.0067	0.0069	0.0101	0.0101
20	0.0043	0.0068	0.0068	0.0068		0.0067	0.0069	0.0101	0.0101
21	0.0043	0.0034	0.0034	0.0034					0.0034
22	0.0127	0.0101	0.0102	0.0103		0.0197	0.0101	0.0101	0.0101
23	0.0043	0.0034	0.0034	0.0034		0.0067	0.0034	0.0034	0.0034
24	0.0127	0.0101	0.0102	0.0103		0.0067	0.0040	0.0040	0.0040
1, 3	0.0048	0.0040	0.0040	0.0038		0.0090	0.0059	0.0059	0.0020
1, 9	0.0034	0.0029	0.0029			0.0032	0.0022	0.0022	
1, 15						0.0056	0.0034	0.0034	
1, 24						0.0098	0.0064	0.0064	0.0095
2, 3						0.0059	0.0034	0.0034	
3, 14	0.0040	0.0034	0.0034			0.0059	0.0034	0.0034	
3, 11						0.0059	0.0034	0.0034	
5, 21						0.0067	0.0034	0.0034	
6, 7	0.0250	0.0203	0.0204	0.0205		0.0314	0.0182	0.0181	0.0189
19, 20		0.0017					0.0033		
3, 6, 7	0.0026	0.0034	0.0034	0.0034		0.0066	0.0057	0.0057	0.0081
6, 19, 20		0.0017							
7, 19, 20		0.0017							
1, 6, 7, 24	0.0039	0.0034	0.0034	0.0034					
6, 7, 19, 20	0.0041	0.0017				0.0057	0.0033	0.0034	0.0034
1, 3, 6, 7, 24						0.0041	0.0032	0.0032	
1, 12, 13, 22, 25	0.0039	0.0034	0.0034	0.0034		0.0053	0.0034	0.0034	0.0034

Estimates of haplotype frequencies for the 25 rare variants in the *MGLL* region obtained by CDMLE (collapsed data maximum likelihood estimation), EML (EM with combinatorially determined list), EM-CDL (EM with CD list) and EM-ATCDL (EM with augmented and trimmed CD list with adaptive threshold) based on *n*=148/*k* pools of *k* individuals each.

Table 2 reports the running times of various algorithms. It can be seen that the EML algorithm takes longer to run than EM-CDL and EM-ATCDL, and is computationally prohibitive (takes longer than 10 hours on an Intel (R) Core (TM) 2 desktop) when the pool size is *k*=4 in both the 25 and 32 loci cases. Both EM-CDL and EM-ATCDL remain computationally feasible when *k*=4. Understandably, EM-CDL is a bit faster to run as no augmentation and trimming is involved.

**Table 2:** Running times of EM algorithms based on different lists

	***MGLL***				***FAAH***		
	**EML**	**EM-CDL**	**EM-ATCDL**		**EML**	**EM-CDL**	**EM-ATCDL**
*k*=1	1.14	0.08	3.68		0.72	0.13	4.57
*k*=2	18.71	0.10	7.05		126.38	0.17	6.78
*k*=4	> 10 h	0.23	7.39		> 10 h	0.13	27.93

Running times in seconds for EML (EM with combinatorially determined list), EM-CDL (EM with CD list) and EM-ATCDL (EM with augmented and trimmed CD list with adaptive threshold) for estimating the haplotype distributions of the 25 rare variants in the *MGLL* region and the 32 rare variants in the *FAAH* region when 148 obese individuals are grouped into pools of various sizes.

To facilitate comparison of estimators in situations similar to those under which the original data were collected, we simulate haplotype data from the *MGLL* region (25 loci) and *FAAH* region (32 loci) according to the haplotype distributions listed as “true” in Tables 3 and 4. These distributions are actually the haplotype distributions estimated using EM-CDL from the individual genotype data of the 148 cases of the CRESCENDO cohort study, but we will treat them as the true distributions in our simulation study. Thus there are only 22 possible haplotypes for the 25 loci case, and 32 haplotypes for the 32 loci case. After generating the haplotypes, we form *n* pools of 2*k* haplotypes each (*n*=100,200; *k*=1,2,3,4) and the resulting pooled genotype data will be treated as the observed data to be used to construct estimates. The results reported in Tables 3 and 4 are based on 100 simulations. The gold standard that we use is the EM-PL estimator, which assumes knowledge of the perfect list (i.e, knowing exactly which *f*(*y*)>0). Because the perfect list is used, the EM algorithm in this case will yield the MLE based on the pooled genotype data. We will not have such knowledge in reality and so our real interest is in comparing the performance of the following estimators: CDMLE (collapsed data MLE), EML (EM with combinatorially determined list), EM-CDL (CD list), EM-ACDL (augmented CD list), EM-ATCDL (augmented and trimmed CD list), and EM-TCDL (CD list with trimming and no augmentation). For removing haplotypes from both the ATCD and TCD lists, we try threshold values from 0.0001 to 0.002 in steps of 0.0001, and select the threshold to yield an estimate of *f*(**0**) as close to ${\hat{f}}_{\text{CD}}(0)$ as possible. Based on the study of Kuk *et al.*[29], ${\hat{f}}_{\text{CD}}(0)$ seems to be a reasonable benchmark to use. In fact, we can see from Tables 3 and 4 that the average of ${\hat{f}}_{\text{CD}}(0)$ (over 100 simulations) is always close to the average of the gold standard ${\hat{f}}_{\text{EM}-\text{PL}}(0)$, and this lends further support to the use of ${\hat{f}}_{\text{CD}}(0)$ as a benchmark. We have simulated data for *k*=1,2,3,4. As the EML algorithm takes too long to run (see Table 2), we compute the EML estimates for *k*=1 and 2 only. To save space, we only report the results of *k*=2 and 4 in Tables 3 and 4. The results for EM-CDL and EM-ACDL are close, and so we table the results of EM-CDL only. In order not to make the tables unduly long, we table only the averages of $\hat{f}(y)$ for those *y* with *f*(*y*)>0, together with the sum of $\hat{f}(y)$ over the remaining *y*’s, as well as the averages over simulations of the sum of squared errors $\underset{y\in \Omega }{\sum }{\left\{\hat{f}(y)-f(y)\right\}}^{2}$, *Ω*={0,1}^*L*^, for the various estimators of *f*(*y*). To supplement Tables 3 and 4, we plot the simulated averages of the sum of squared errors against pool size *k* for all 7 estimators, including EM-PL.

**Table 3:** Average estimates of haplotype frequencies for a 25 loci case

			***k=2***						***k=4***			
**Position of ‘1’**	**TRUE**		**CDMLE**	**EML**	**EM- CDL**	**EM-ATCDL**	**EM-TCDL**		**CDMLE**	**EM-CDL**	**EM-ATCDL**	**EM-TCDL**
						**(a)*n=100***						
None	0.7995		0.7973	0.8279	0.8283	0.8003	0.8067		0.7961	0.8535	0.7957	0.8119
			(0.0232)	(0.0169)	(0.0170)	(0.0215)	(0.0192)		(0.0204)	(0.0118)	(0.0179)	(0.0144)
1	0.0509		0.0508	0.0412	0.0412	0.0477	0.0477		0.0502	0.0344	0.0457	0.0494
			(0.0155)	(0.0115)	(0.0115)	(0.0119)	(0.0112)		(0.0152)	(0.0083)	(0.0093)	(0.0086)
2	0.0034		0.0036	0.0019	0.0019	0.0031	0.0024		0.0032	0.0008	0.0028	0.0020
			(0.0040)	(0.0022)	(0.0022)	(0.0025)	(0.0027)		(0.0044)	(0.0012)	(0.0023)	(0.0029)
3	0.0436		0.0441	0.0353	0.0353	0.0425	0.0408		0.0435	0.0277	0.0396	0.0411
			(0.0139)	(0.0104)	(0.0104)	(0.0112)	(0.0107)		(0.0144)	(0.0077)	(0.0097)	(0.0080)
5	0.0034		0.0035	0.0019	0.0019	0.0031	0.0027		0.0031	0.0008	0.0028	0.0015
			(0.0039)	(0.0021)	(0.0021)	(0.0028)	(0.0029)		(0.0043)	(0.0011)	(0.0017)	(0.0024)
6	0.0034		0.0027	0.0017	0.0016	0.0029	0.0022		0.0038	0.0013	0.0030	0.0019
			(0.0035)	(0.0021)	(0.0021)	(0.0028)	(0.0030)		(0.0050)	(0.0017)	(0.0022)	(0.0026)
9	0.0073		0.0092	0.0056	0.0056	0.0085	0.0087		0.0073	0.0029	0.0074	0.0079
			(0.0065)	(0.0039)	(0.0039)	(0.0046)	(0.0052)		(0.0066)	(0.0026)	(0.0045)	(0.0062)
11	0.0034		0.0041	0.0022	0.0022	0.0036	0.0029		0.0032	0.0008	0.0027	0.0016
			(0.0048)	(0.0025)	(0.0025)	(0.0029)	(0.0031)		(0.0046)	(0.0013)	(0.0021)	(0.0025)
15	0.0034		0.0039	0.0021	0.0021	0.0032	0.0026		0.0034	0.0009	0.0029	0.0017
			(0.0052)	(0.0028)	(0.0028)	(0.0032)	(0.0033)		(0.0050)	(0.0013)	(0.0021)	(0.0025)
19	0.0068		0.0069	0.0039	0.0039	0.0061	0.0055		0.0075	0.0022	0.0058	0.0051
			(0.0056)	(0.0031)	(0.0031)	(0.0040)	(0.0044)		(0.0066)	(0.0020)	(0.0031)	(0.0042)
20	0.0068		0.0073	0.0041	0.0041	0.0061	0.0058		0.0080	0.0023	0.0057	0.0051
			(0.0060)	(0.0035)	(0.0035)	(0.0042)	(0.0045)		(0.0065)	(0.0020)	(0.0028)	(0.0040)
21	0.0034		0.0038	0.0020	0.0020	0.0032	0.0029		0.0041	0.0011	0.0033	0.0022
			(0.0041)	(0.0022)	(0.0022)	(0.0027)	(0.0031)		(0.0051)	(0.0014)	(0.0023)	(0.0028)
22	0.0102		0.0117	0.0070	0.0070	0.0095	0.0099		0.0110	0.0043	0.0096	0.0091
			(0.0075)	(0.0047)	(0.0047)	(0.0053)	(0.0054)		(0.0085)	(0.0033)	(0.0046)	(0.0056)
23	0.0034		0.0032	0.0018	0.0018	0.0028	0.0024		0.0038	0.0010	0.0029	0.0019
			(0.0039)	(0.0023)	(0.0023)	(0.0028)	(0.0030)		(0.0045)	(0.0012)	(0.0021)	(0.0026)
24	0.0102		0.0096	0.0060	0.0060	0.0095	0.0098		0.0114	0.0043	0.0098	0.0118
			(0.0065)	(0.0041)	(0.0041)	(0.0051)	(0.0057)		(0.0076)	(0.0028)	(0.0047)	(0.0057)
1, 3	0.0040		0.0048	0.0039	0.0039	0.0045	0.0043		0.0071	0.0049	0.0061	0.0052
			(0.0049)	(0.0037)	(0.0037)	(0.0041)	(0.0040)		(0.0070)	(0.0040)	(0.0046)	(0.0048)
1, 9	0.0029		0.0030	0.0021	0.0021	0.0023	0.0018		0.0051	0.0023	0.0028	0.0017
			(0.0035)	(0.0024)	(0.0024)	(0.0033)	(0.0033)		(0.0055)	(0.0021)	(0.0031)	(0.0032)
6, 7	0.0204		0.0210	0.0150	0.0148	0.0215	0.0195		0.0203	0.0104	0.0210	0.0219
			(0.0098)	(0.0072)	(0.0072)	(0.0075)	(0.0077)		(0.0105)	(0.0043)	(0.0068)	(0.0060)
3, 14	0.0034		0.0035	0.0021	0.0021	0.0020	0.0028		0.0031	0.0012	0.0015	0.0021
			(0.0037)	(0.0022)	(0.0022)	(0.0032)	(0.0031)		(0.0038)	(0.0015)	(0.0024)	(0.0026)
3, 6, 7	0.0034		0.0037	0.0028	0.0029	0.0025	0.0030		0.0047	0.0028	0.0022	0.0016
			(0.0047)	(0.0036)	(0.0036)	(0.0044)	(0.0047)		(0.0053)	(0.0025)	(0.0033)	(0.0035)
1, 6, 7, 24	0.0034		0.0031	0.0021	0.0021	0.0006	0.0006		0.0036	0.0018	0.0006	0.0000
			(0.0033)	(0.0023)	(0.0023)	(0.0023)	(0.0021)		(0.0037)	(0.0018)	(0.0017)	(0.0004)
1, 12, 13, 22, 25	0.0034		0.0037	0.0024	0.0024	0.0026	0.0029		0.0038	0.0016	0.0005	0.0025
			(0.0038)	(0.0025)	(0.0025)	(0.0031)	(0.0029)		(0.0035)	(0.0015)	(0.0018)	(0.0027)
Sum of remaining			0.0376	0.0248	0.0248	0.0117	0.0124		0.0893	0.0366	0.0255	0.0109
haplotype probabilities												
Sum of probabilities			0.0247	0.0241	0.0244	0.0218	0.0286		0.0296	0.0285	0.0179	0.0367
of missed haplotypes												
Sum of squared errors			0.00166	0.00186	0.00189	0.00110	0.00106		0.00201	0.00415	0.00091	0.00089
Length of list			26.77	116.28	26.77	19.06	18.25		45.23	45.23	25.38	15.62
SD of length			(3.26)	(81.30)	(3.26)	(3.03)	(2.18)		(3.94)	(3.94)	(4.78)	(2.40)
						**(a)*n=200***						
None	0.7995		0.7979	0.8248	0.8250	0.7981	0.8009		0.7990	0.8451	0.7970	0.8040
			(0.0150)	(0.0117)	(0.0117)	(0.0148)	(0.0132)		(0.0154)	(0.0086)	(0.0133)	(0.0125)
1	0.0509		0.0514	0.0433	0.0433	0.0492	0.0503		0.0502	0.0387	0.0462	0.0507
			(0.0103)	(0.0082)	(0.0082)	(0.0089)	(0.0088)		(0.0121)	(0.0069)	(0.0077)	(0.0080)
2	0.0034		0.0035	0.0020	0.0020	0.0033	0.0031		0.0037	0.0011	0.0030	0.0024
			(0.0032)	(0.0018)	(0.0018)	(0.0024)	(0.0026)		(0.0035)	(0.0011)	(0.0013)	(0.0021)
3	0.0436		0.0430	0.0362	0.0362	0.0435	0.0426		0.0441	0.0322	0.0406	0.0426
			(0.0092)	(0.0075)	(0.0075)	(0.0082)	(0.0074)		(0.0105)	(0.0054)	(0.0071)	(0.0065)
5	0.0034		0.0033	0.0018	0.0018	0.0032	0.0028		0.0034	0.0011	0.0030	0.0023
			(0.0028)	(0.0016)	(0.0016)	(0.0020)	(0.0023)		(0.0035)	(0.0011)	(0.0013)	(0.0021)
6	0.0034		0.0038	0.0023	0.0023	0.0033	0.0031		0.0033	0.0014	0.0028	0.0022
			(0.0031)	(0.0019)	(0.0020)	(0.0021)	(0.0025)		(0.0035)	(0.0013)	(0.0014)	(0.0019)
9	0.0073		0.0080	0.0054	0.0054	0.0079	0.0088		0.0081	0.0036	0.0070	0.0092
			(0.0038)	(0.0026)	(0.0026)	(0.0032)	(0.0037)		(0.0043)	(0.0019)	(0.0026)	(0.0031)
11	0.0034		0.0032	0.0018	0.0018	0.0030	0.0027		0.0032	0.0010	0.0029	0.0023
			(0.0026)	(0.0016)	(0.0016)	(0.0022)	(0.0024)		(0.0031)	(0.0011)	(0.0015)	(0.0021)
15	0.0034		0.0035	0.0019	0.0019	0.0030	0.0028		0.0038	0.0012	0.0031	0.0028
			(0.0033)	(0.0018)	(0.0018)	(0.0023)	(0.0025)		(0.0031)	(0.0010)	(0.0015)	(0.0021)
19	0.0068		0.0063	0.0039	0.0039	0.0062	0.0061		0.0066	0.0026	0.0056	0.0057
			(0.0039)	(0.0025)	(0.0025)	(0.0029)	(0.0034)		(0.0041)	(0.0015)	(0.0018)	(0.0026)
20	0.0068		0.0068	0.0042	0.0042	0.0062	0.0063		0.0063	0.0026	0.0054	0.0059
			(0.0039)	(0.0025)	(0.0025)	(0.0028)	(0.0030)		(0.0038)	(0.0015)	(0.0022)	(0.0026)
21	0.0034		0.0038	0.0022	0.0022	0.0035	0.0032		0.0037	0.0012	0.0031	0.0025
			(0.0035)	(0.0020)	(0.0020)	(0.0023)	(0.0026)		(0.0034)	(0.0011)	(0.0015)	(0.0021)
22	0.0102		0.0105	0.0071	0.0071	0.0097	0.0103		0.0112	0.0052	0.0086	0.0098
			(0.0058)	(0.0037)	(0.0037)	(0.0041)	(0.0041)		(0.0052)	(0.0022)	(0.0030)	(0.0029)
23	0.0034		0.0039	0.0022	0.0022	0.0031	0.0030		0.0035	0.0011	0.0030	0.0025
			(0.0027)	(0.0015)	(0.0016)	(0.0021)	(0.0022)		(0.0030)	(0.0010)	(0.0015)	(0.0021)
24	0.0102		0.0105	0.0069	0.0069	0.0107	0.0114		0.0106	0.0049	0.0088	0.0115
			(0.0050)	(0.0033)	(0.0033)	(0.0043)	(0.0042)		(0.0059)	(0.0023)	(0.0036)	(0.0043)
1, 3	0.0040		0.0046	0.0037	0.0037	0.0041	0.0040		0.0052	0.0041	0.0049	0.0047
			(0.0041)	(0.0032)	(0.0032)	(0.0033)	(0.0034)		(0.0048)	(0.0029)	(0.0030)	(0.0031)
1, 9	0.0029		0.0036	0.0026	0.0026	0.0030	0.0022		0.0030	0.0018	0.0027	0.0010
			(0.0027)	(0.0019)	(0.0019)	(0.0026)	(0.0028)		(0.0031)	(0.0015)	(0.0022)	(0.0020)
6, 7	0.0204		0.0191	0.0148	0.0146	0.0215	0.0199		0.0206	0.0124	0.0212	0.0223
			(0.0067)	(0.0048)	(0.0047)	(0.0056)	(0.0054)		(0.0073)	(0.0037)	(0.0048)	(0.0046)
3, 14	0.0034		0.0030	0.0019	0.0019	0.0020	0.0027		0.0036	0.0015	0.0025	0.0029
			(0.0029)	(0.0018)	(0.0018)	(0.0026)	(0.0025)		(0.0026)	(0.0011)	(0.0020)	(0.0018)
3, 6, 7	0.0034		0.0039	0.0029	0.0029	0.0021	0.0027		0.0038	0.0025	0.0024	0.0019
			(0.0035)	(0.0025)	(0.0025)	(0.0029)	(0.0031)		(0.0041)	(0.0022)	(0.0028)	(0.0029)
1, 6, 7, 24	0.0034		0.0039	0.0028	0.0028	0.0007	0.0006		0.0039	0.0020	0.0002	0.0000
			(0.0025)	(0.0019)	(0.0019)	(0.0019)	(0.0018)		(0.0026)	(0.0014)	(0.0009)	(0.0002)
1, 12, 13, 22, 25	0.0034		0.0035	0.0025	0.0024	0.0027	0.0032		0.0033	0.0017	0.0004	0.0031
			(0.0022)	(0.0016)	(0.0017)	(0.0023)	(0.0023)		(0.0026)	(0.0013)	(0.0014)	(0.0020)
Sum of remaining			0.0340	0.0227	0.0227	0.0102	0.0074		0.0703	0.0310	0.0255	0.0076
haplotype probabilities												
Sum of probabilities			0.0103	0.0097	0.0100	0.0132	0.0173		0.0131	0.0118	0.0111	0.0200
of missed haplotypes												
Sum of squared errors			0.00077	0.00125	0.00126	0.00059	0.00054		0.00101	0.00281	0.00055	0.00048
Length of list			39.65	152.30	39.65	22.08	19.63		71.24	71.24	29.29	19.81
SD of length			(3.90)	(71.65)	(3.90)	(4.20)	(3.70)		(5.02)	(5.02)	(5.82)	(5.22)

Average estimates of haplotype frequencies for a 25 loci case based on 100 simulations of *n* pools of *k* individuals each using CDMLE (collapsed data MLE), EML (EM with combinatorially determined list), EM-CDL (EM with CD list), EM-ATCDL (augmented and trimmed CD list) and EM-TCDL (CD list with trimming and no augmentation), with standard errors in parentheses.

**Table 4:** Average estimates of haplotype frequencies for a 32 loci case

			***k=2***						***k=4***			
**Position of ‘1’**	**TRUE**		**CDMLE**	**EML**	**EM- CDL**	**EM-ATCDL**	**EM-TCDL**		**CDMLE**	**EM-CDL**	**EM-ATCDL**	**EM-TCDL**
						**(a)*n=100***						
None	0.7995		0.7979	0.8248	0.8250	0.7981	0.8009		0.7990	0.8451	0.7970	0.8040
			(0.0150)	(0.0117)	(0.0117)	(0.0148)	(0.0132)		(0.0154)	(0.0086)	(0.0133)	(0.0125)
1	0.0509		0.0514	0.0433	0.0433	0.0492	0.0503		0.0502	0.0387	0.0462	0.0507
			(0.0103)	(0.0082)	(0.0082)	(0.0089)	(0.0088)		(0.0121)	(0.0069)	(0.0077)	(0.0080)
2	0.0034		0.0035	0.0020	0.0020	0.0033	0.0031		0.0037	0.0011	0.0030	0.0024
			(0.0032)	(0.0018)	(0.0018)	(0.0024)	(0.0026)		(0.0035)	(0.0011)	(0.0013)	(0.0021)
3	0.0436		0.0430	0.0362	0.0362	0.0435	0.0426		0.0441	0.0322	0.0406	0.0426
			(0.0092)	(0.0075)	(0.0075)	(0.0082)	(0.0074)		(0.0105)	(0.0054)	(0.0071)	(0.0065)
5	0.0034		0.0033	0.0018	0.0018	0.0032	0.0028		0.0034	0.0011	0.0030	0.0023
			(0.0028)	(0.0016)	(0.0016)	(0.0020)	(0.0023)		(0.0035)	(0.0011)	(0.0013)	(0.0021)
6	0.0034		0.0038	0.0023	0.0023	0.0033	0.0031		0.0033	0.0014	0.0028	0.0022
			(0.0031)	(0.0019)	(0.0020)	(0.0021)	(0.0025)		(0.0035)	(0.0013)	(0.0014)	(0.0019)
9	0.0073		0.0080	0.0054	0.0054	0.0079	0.0088		0.0081	0.0036	0.0070	0.0092
			(0.0038)	(0.0026)	(0.0026)	(0.0032)	(0.0037)		(0.0043)	(0.0019)	(0.0026)	(0.0031)
11	0.0034		0.0032	0.0018	0.0018	0.0030	0.0027		0.0032	0.0010	0.0029	0.0023
			(0.0026)	(0.0016)	(0.0016)	(0.0022)	(0.0024)		(0.0031)	(0.0011)	(0.0015)	(0.0021)
15	0.0034		0.0035	0.0019	0.0019	0.0030	0.0028		0.0038	0.0012	0.0031	0.0028
			(0.0033)	(0.0018)	(0.0018)	(0.0023)	(0.0025)		(0.0031)	(0.0010)	(0.0015)	(0.0021)
19	0.0068		0.0063	0.0039	0.0039	0.0062	0.0061		0.0066	0.0026	0.0056	0.0057
			(0.0039)	(0.0025)	(0.0025)	(0.0029)	(0.0034)		(0.0041)	(0.0015)	(0.0018)	(0.0026)
20	0.0068		0.0068	0.0042	0.0042	0.0062	0.0063		0.0063	0.0026	0.0054	0.0059
			(0.0039)	(0.0025)	(0.0025)	(0.0028)	(0.0030)		(0.0038)	(0.0015)	(0.0022)	(0.0026)
21	0.0034		0.0038	0.0022	0.0022	0.0035	0.0032		0.0037	0.0012	0.0031	0.0025
			(0.0035)	(0.0020)	(0.0020)	(0.0023)	(0.0026)		(0.0034)	(0.0011)	(0.0015)	(0.0021)
22	0.0102		0.0105	0.0071	0.0071	0.0097	0.0103		0.0112	0.0052	0.0086	0.0098
			(0.0058)	(0.0037)	(0.0037)	(0.0041)	(0.0041)		(0.0052)	(0.0022)	(0.0030)	(0.0029)
23	0.0034		0.0039	0.0022	0.0022	0.0031	0.0030		0.0035	0.0011	0.0030	0.0025
			(0.0027)	(0.0015)	(0.0016)	(0.0021)	(0.0022)		(0.0030)	(0.0010)	(0.0015)	(0.0021)
24	0.0102		0.0105	0.0069	0.0069	0.0107	0.0114		0.0106	0.0049	0.0088	0.0115
			(0.0050)	(0.0033)	(0.0033)	(0.0043)	(0.0042)		(0.0059)	(0.0023)	(0.0036)	(0.0043)
1, 3	0.0040		0.0046	0.0037	0.0037	0.0041	0.0040		0.0052	0.0041	0.0049	0.0047
			(0.0041)	(0.0032)	(0.0032)	(0.0033)	(0.0034)		(0.0048)	(0.0029)	(0.0030)	(0.0031)
1, 9	0.0029		0.0036	0.0026	0.0026	0.0030	0.0022		0.0030	0.0018	0.0027	0.0010
			(0.0027)	(0.0019)	(0.0019)	(0.0026)	(0.0028)		(0.0031)	(0.0015)	(0.0022)	(0.0020)
6, 7	0.0204		0.0191	0.0148	0.0146	0.0215	0.0199		0.0206	0.0124	0.0212	0.0223
			(0.0067)	(0.0048)	(0.0047)	(0.0056)	(0.0054)		(0.0073)	(0.0037)	(0.0048)	(0.0046)
3, 14	0.0034		0.0030	0.0019	0.0019	0.0020	0.0027		0.0036	0.0015	0.0025	0.0029
			(0.0029)	(0.0018)	(0.0018)	(0.0026)	(0.0025)		(0.0026)	(0.0011)	(0.0020)	(0.0018)
3, 6, 7	0.0034		0.0039	0.0029	0.0029	0.0021	0.0027		0.0038	0.0025	0.0024	0.0019
			(0.0035)	(0.0025)	(0.0025)	(0.0029)	(0.0031)		(0.0041)	(0.0022)	(0.0028)	(0.0029)
1, 6, 7, 24	0.0034		0.0039	0.0028	0.0028	0.0007	0.0006		0.0039	0.0020	0.0002	0.0000
			(0.0025)	(0.0019)	(0.0019)	(0.0019)	(0.0018)		(0.0026)	(0.0014)	(0.0009)	(0.0002)
1, 12, 13, 22, 25	0.0034		0.0035	0.0025	0.0024	0.0027	0.0032		0.0033	0.0017	0.0004	0.0031
			(0.0022)	(0.0016)	(0.0017)	(0.0023)	(0.0023)		(0.0026)	(0.0013)	(0.0014)	(0.0020)
Sum of remaining			0.0340	0.0227	0.0227	0.0102	0.0074		0.0703	0.0310	0.0255	0.0076
haplotype probabilities												
Sum of probabilities			0.0103	0.0097	0.0100	0.0132	0.0173		0.0131	0.0118	0.0111	0.0200
of missed haplotypes												
Sum of squared errors			0.00077	0.00125	0.00126	0.00059	0.00054		0.00101	0.00281	0.00055	0.00048
Length of list			39.65	152.30	39.65	22.08	19.63		71.24	71.24	29.29	19.81
SD of length			(3.90)	(71.65)	(3.90)	(4.20)	(3.70)		(5.02)	(5.02)	(5.82)	(5.22)
						**(b)*n=200***						
None	0.7113		0.7108	0.7674	0.7677	0.7115	0.7219		0.7110	0.8075	0.7109	0.7374
			(0.0202)	(0.0132)	(0.0132)	(0.0197)	(0.0161)		(0.0232)	(0.0119)	(0.0192)	(0.0147)
1	0.0034		0.0031	0.0013	0.0013	0.0032	0.0022		0.0034	0.0005	0.0031	0.0012
			(0.0032)	(0.0014)	(0.0014)	(0.0022)	(0.0025)		(0.0054)	(0.0007)	(0.0015)	(0.0018)
3	0.0034		0.0039	0.0016	0.0016	0.0032	0.0028		0.0036	0.0005	0.0030	0.0017
			(0.0034)	(0.0014)	(0.0014)	(0.0022)	(0.0025)		(0.0047)	(0.0007)	(0.0014)	(0.0022)
5	0.0034		0.0037	0.0016	0.0016	0.0035	0.0029		0.0036	0.0006	0.0032	0.0017
			(0.0035)	(0.0015)	(0.0015)	(0.0022)	(0.0027)		(0.0049)	(0.0007)	(0.0013)	(0.0022)
7	0.0068		0.0066	0.0038	0.0041	0.0061	0.0064		0.0065	0.0027	0.0058	0.0061
			(0.0048)	(0.0024)	(0.0028)	(0.0030)	(0.0040)		(0.0070)	(0.0024)	(0.0024)	(0.0053)
9	0.0034		0.0037	0.0015	0.0015	0.0034	0.0029		0.0038	0.0006	0.0031	0.0016
			(0.0038)	(0.0016)	(0.0016)	(0.0024)	(0.0029)		(0.0049)	(0.0008)	(0.0015)	(0.0022)
10	0.0102		0.0098	0.0050	0.0050	0.0087	0.0093		0.0103	0.0026	0.0088	0.0085
			(0.0059)	(0.0029)	(0.0030)	(0.0035)	(0.0035)		(0.0072)	(0.0017)	(0.0026)	(0.0042)
11	0.0034		0.0038	0.0016	0.0016	0.0032	0.0029		0.0027	0.0004	0.0031	0.0010
			(0.0030)	(0.0013)	(0.0013)	(0.0023)	(0.0026)		(0.0044)	(0.0007)	(0.0014)	(0.0019)
14	0.0034		0.0034	0.0014	0.0014	0.0031	0.0023		0.0030	0.0004	0.0029	0.0013
			(0.0037)	(0.0016)	(0.0016)	(0.0022)	(0.0027)		(0.0048)	(0.0007)	(0.0015)	(0.0021)
17	0.0034		0.0033	0.0014	0.0014	0.0029	0.0023		0.0031	0.0005	0.0028	0.0014
			(0.0035)	(0.0014)	(0.0014)	(0.0021)	(0.0024)		(0.0047)	(0.0008)	(0.0015)	(0.0021)
20	0.0034		0.0037	0.0015	0.0015	0.0030	0.0025		0.0028	0.0004	0.0029	0.0014
			(0.0035)	(0.0014)	(0.0014)	(0.0019)	(0.0025)		(0.0042)	(0.0006)	(0.0014)	(0.0021)
21	0.0264		0.0251	0.0164	0.0164	0.0252	0.0252		0.0266	0.0117	0.0257	0.0260
			(0.0089)	(0.0051)	(0.0051)	(0.0064)	(0.0063)		(0.0135)	(0.0040)	(0.0055)	(0.0056)
22	0.0068		0.0074	0.0040	0.0040	0.0095	0.0099		0.0067	0.0016	0.0089	0.0074
			(0.0055)	(0.0029)	(0.0029)	(0.0040)	(0.0051)		(0.0067)	(0.0017)	(0.0038)	(0.0062)
24	0.0306		0.0307	0.0223	0.0234	0.0289	0.0295		0.0297	0.0194	0.0273	0.0291
			(0.0096)	(0.0066)	(0.0066)	(0.0070)	(0.0074)		(0.0146)	(0.0049)	(0.0058)	(0.0061)
25	0.0136		0.0138	0.0075	0.0075	0.0129	0.0129		0.0155	0.0047	0.0139	0.0125
			(0.0072)	(0.0036)	(0.0036)	(0.0040)	(0.0036)		(0.0100)	(0.0029)	(0.0042)	(0.0051)
26	0.0034		0.0033	0.0013	0.0013	0.0029	0.0023		0.0037	0.0005	0.0031	0.0014
			(0.0035)	(0.0014)	(0.0014)	(0.0024)	(0.0026)		(0.0052)	(0.0007)	(0.0014)	(0.0021)
28	0.0675		0.0661	0.0487	0.0488	0.0615	0.0640		0.0668	0.0390	0.0614	0.0629
			(0.0136)	(0.0089)	(0.0089)	(0.0107)	(0.0105)		(0.0162)	(0.0075)	(0.0090)	(0.0086)
30	0.0036		0.0032	0.0017	0.0017	0.0070	0.0064		0.0043	0.0009	0.0077	0.0060
			(0.0029)	(0.0015)	(0.0015)	(0.0039)	(0.0056)		(0.0053)	(0.0011)	(0.0040)	(0.0067)
31	0.0034		0.0037	0.0016	0.0016	0.0031	0.0025		0.0031	0.0004	0.0030	0.0014
			(0.0037)	(0.0016)	(0.0016)	(0.0021)	(0.0025)		(0.0044)	(0.0006)	(0.0013)	(0.0020)
32	0.0038		0.0034	0.0016	0.0016	0.0045	0.0041		0.0041	0.0007	0.0042	0.0029
			(0.0032)	(0.0015)	(0.0016)	(0.0029)	(0.0037)		(0.0060)	(0.0010)	(0.0021)	(0.0038)
2, 25	0.0034		0.0034	0.0015	0.0015	0.0021	0.0026		0.0036	0.0006	0.0013	0.0018
			(0.0033)	(0.0015)	(0.0015)	(0.0029)	(0.0028)		(0.0048)	(0.0008)	(0.0020)	(0.0023)
7, 24	0.0510		0.0507	0.0403	0.0386	0.0525	0.0530		0.0523	0.0319	0.0524	0.0519
			(0.0120)	(0.0079)	(0.0078)	(0.0094)	(0.0084)		(0.0139)	(0.0063)	(0.0089)	(0.0064)
12, 13	0.0034		0.0031	0.0013	0.0013	0.0016	0.0023		0.0029	0.0005	0.0011	0.0014
			(0.0033)	(0.0015)	(0.0015)	(0.0025)	(0.0026)		(0.0049)	(0.0009)	(0.0020)	(0.0022)
21, 23	0.0034		0.0031	0.0015	0.0015	0.0018	0.0023		0.0035	0.0007	0.0016	0.0019
			(0.0035)	(0.0016)	(0.0016)	(0.0027)	(0.0026)		(0.0042)	(0.0009)	(0.0023)	(0.0023)
21, 28	0.0009		0.0031	0.0021	0.0021	0.0022	0.0019		0.0035	0.0016	0.0015	0.0010
			(0.0038)	(0.0022)	(0.0022)	(0.0027)	(0.0030)		(0.0050)	(0.0017)	(0.0023)	(0.0027)
21, 30	0.0033		0.0035	0.0018	0.0018	0.0022	0.0018		0.0035	0.0008	0.0016	0.0012
			(0.0033)	(0.0016)	(0.0016)	(0.0027)	(0.0027)		(0.0044)	(0.0010)	(0.0024)	(0.0023)
22, 30	0.0034		0.0029	0.0014	0.0013	0.0017	0.0017		0.0037	0.0007	0.0014	0.0014
			(0.0031)	(0.0015)	(0.0015)	(0.0024)	(0.0027)		(0.0051)	(0.0009)	(0.0021)	(0.0022)
24, 28	0.0034		0.0042	0.0034	0.0033	0.0036	0.0035		0.0064	0.0036	0.0037	0.0040
			(0.0048)	(0.0030)	(0.0030)	(0.0032)	(0.0034)		(0.0072)	(0.0028)	(0.0029)	(0.0036)
28, 32	0.0030		0.0034	0.0019	0.0019	0.0022	0.0019		0.0033	0.0011	0.0018	0.0018
			(0.0033)	(0.0018)	(0.0019)	(0.0028)	(0.0028)		(0.0037)	(0.0011)	(0.0021)	(0.0026)
4, 7, 24	0.0034		0.0028	0.0015	0.0016	0.0013	0.0024		0.0028	0.0008	0.0008	0.0019
			(0.0025)	(0.0014)	(0.0014)	(0.0022)	(0.0022)		(0.0027)	(0.0008)	(0.0016)	(0.0019)
7, 22, 24	0.0034		0.0042	0.0025	0.0026	0.0019	0.0011		0.0029	0.0015	0.0018	0.0012
			(0.0036)	(0.0022)	(0.0021)	(0.0028)	(0.0024)		(0.0034)	(0.0013)	(0.0024)	(0.0025)
7, 24, 30	0.0034		0.0034	0.0023	0.0023	0.0019	0.0018		0.0033	0.0014	0.0018	0.0018
			(0.0032)	(0.0022)	(0.0022)	(0.0031)	(0.0031)		(0.0035)	(0.0013)	(0.0024)	(0.0029)
Sum of remaining			0.0828	0.0454	0.0453	0.0178	0.0083		0.2194	0.0592	0.0243	0.0160
haplotype probabilities												
Sum of probabilities			0.0278	0.0265	0.0271	0.0263	0.0384		0.0474	0.0462	0.0222	0.0553
of missed haplotypes												
Sum of squared errors			0.00161	0.00451	0.00456	0.00103	0.00100		0.00334	0.01152	0.00092	0.00152
Length of list			62.39	159.68	62.39	30.60	23.57		106.91	106.91	36.11	21.92
SD of length			(4.88)	(27.84)	(4.88)	(6.04)	(4.22)		(6.41)	(6.41)	(7.12)	(5.11)

Average estimates of haplotype frequencies for a 32 loci case based on 100 simulations of *n* pools of *k* individuals each using CDMLE (collapsed data MLE), EML (EM with combinatorially determined list), EM-CDL (EM with CD list), EM-ATCDL (augmented and trimmed CD list) and EM-TCDL (CD list with trimming and no augmentation), with standard errors in parentheses.

It can be seen from Tables 3 and 4 that EM-CDL overestimated the frequency *f*(**0**) of the ancestral haplotype quite severely, and it has the largest sum of squared error among all the estimators. The performance of EML is very similar to that of EM-CDL (both unsatisfactory) but the computational cost is much higher. It suffers from assigning small probabilities to too many false haplotypes. For example, for the 25 loci case with *n*=100, *k*=2, the EML list on the average contains 116 haplotypes even though the true distribution is concentrated on 22 haplotypes. The total probability that the EML estimator attaches to haplotypes outside of the true 22 is only 0.0248 on the average. This foretells the need for trimming which is a point we will come back to later.

**Figure 1:**
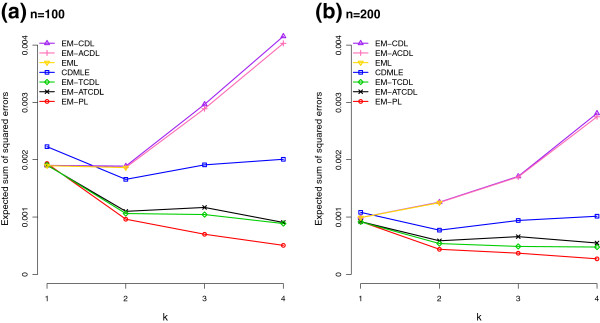
**Expected sum of squared errors of various haplotype frequency estimators for a 25 loci case.** Expected sum of squared errors of various haplotype frequency estimators (EM-CDL: EM with CD list; EM-ACDL: augmented CD list; EML: EM with combinatorially determined list; CDMLE: collapsed data MLE; EM-TCDL: CD list with trimming and no augmentation; EM-ATCDL: augmented and trimmed CD list; EM-PL: EM with perfect list) based on 100 simulations of **(a)***n*=100 and **(b)***n*=200 pools of *k* individuals each when the true haplotype distribution over 25 loci is as given in Table 3.

**Figure 2:**
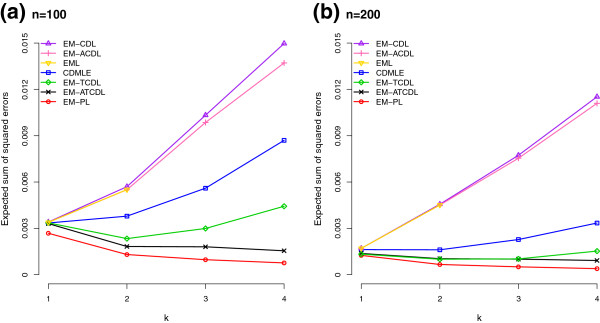
**Expected sum of squared errors of various haplotype frequency estimators for a 32 loci case.** Expected sum of squared errors of various haplotype frequency estimators (EM-CDL: EM with CD list; EM-ACDL: augmented CD list; EML: EM with combinatorially determined list; CDMLE: collapsed data MLE; EM-TCDL: CD list with trimming and no augmentation; EM-ATCDL: augmented and trimmed CD list; EM-PL: EM with perfect list) based on 100 simulations of **(a)***n*=100 and **(b)***n*=200 pools of *k* individuals each when the true haplotype distribution over 32 loci is as given in Table 4.

Augmenting the CD list did not help much as the results for EM-ACDL are almost the same as that of EM-CDL when *n*=200, and only slightly better when *n*=100 (not shown in the tables, but we can see this from Figures 1 and 2). Trimming in addition to augmenting the CD list improved things a lot, as demonstrated by the good results of EM-ATCDL in both Tables 3 and 4. From Figures 1 and 2, we can see that EM-ATCDL is clearly the best estimator among those considered, other than the perfect list estimator which is not a legitimate estimator. Since augmenting alone did not improve results much, but trimming in addition to augmenting did, we were curious to see whether trimming alone would work or not. As expected, we can see from Tables 3 and 4 that the TCD list (trimming without augmentation) is on the average shorter than the ATCD list. Consequently, the TCD list will miss more true haplotypes, and the sum of probabilities of the missed haplotypes is higher for EM-TCDL than for EM-ATCDL, and more so for the 32 loci case and when the number of pools is 100 rather than 200. In particular, the sum of probabilities of the missed haplotypes for the 32 loci case with *k*=4 is 0.0798 (after averaging over simulations) when *n*=100, and improves slightly to 0.0553 when *n*=200. The corresponding figures for EM-ATCDL are 0.0328 and 0.0222. In terms of sum of squared errors, EM-TCDL is also inferior to EM-ATCDL for the 32 loci case, particularly when *n*=100.

The collapsed data MLE advocated by Kuk *et al.*[29] behaves very similarly to the gold standard EM-PL estimator in terms of bias or expected value, but it suffers from having a larger variance, especially for larger pool size. In contrast, the EM-CDL estimates have small variance but large bias. By benchmarking against CDMLE, the EM-ATCDL estimates have smaller bias than EM-CDL and smaller variance than CDMLE. The main advantage of the collapsed data MLE is its simplicity and small bias. As shown by Kuk *et al.*[29], the loss in efficiency due to collapsing the pooled genotype data locus-wise to just “0” and “ ≥1” is not large for small pool size (especially when *k*=1 which corresponds to individual genotype data) and rare alleles, but it is better to use EM-ATCDL if *k*≥2.

**Figure 3:**
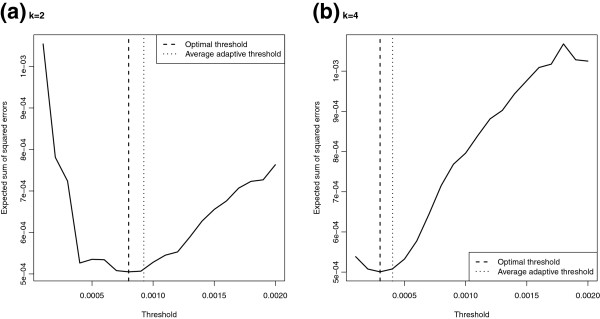
**Expected sum of squared errors of the EM-ATCDL estimator with fixed threshold (25 loci case).** Expected sum of squared errors of the EM-ATCDL estimator for various choices of the threshold based on 100 simulations of *n*=200 pools of **(a)***k*=2 and **(b)***k*=4 individuals each when the true haplotype distribution over 25 loci is as given in Table 3; optimal threshold: the threshold obtained by minimizing the averaged sum of squared errors; average adaptive threshold: adaptively chosen thresholds obtained by minimizing the distance between $\hat{f}(0)$ and *f*(**0**) over the grid 0.0001 to 0.002 in steps of 0.0001.

**Figure 4:**
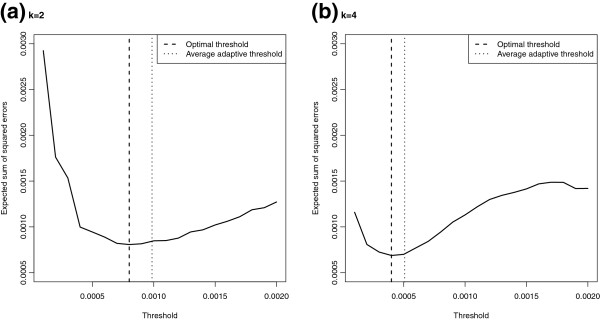
**Expected sum of squared errors of the EM-ATCDL estimator with fixed threshold (32 loci case).** Expected sum of squared errors of the EM-ATCDL estimator for various choices of the threshold based on 100 simulations of *n*=200 pools of **(a)***k*=2 and **(b)***k*=4 individuals each when the true haplotype distribution over 32 loci is as given in Table 4; optimal threshold: the threshold obtained by minimizing the averaged sum of squared errors; average adaptive threshold: adaptively chosen thresholds obtained by minimizing the distance between $\hat{f}(0)$ and *f*(**0**) over the grid 0.0001 to 0.002 in steps of 0.0001.

To further see if our benchmarking method of determining the threshold for the removal of haplotypes is reasonable or not, we also compute the EM-ATCDL estimates based on fixed threshold in our simulation study to find out which threshold is “optimal”. Figures 3 and 4 depict the averages of the sum of squared errors $\underset{y\in \Omega }{\sum }{\left\{\hat{f}(y)-f(y)\right\}}^{2}$, *Ω*={0,1}^*L*^, over 100 simulations for the EM-ATCDL estimates $\hat{f}(y)$ as a function of the threshold value. The position of the “optimal” threshold which minimizes that averaged sum of squared errors is depicted by the vertical dashed line, whereas the average of the adaptively chosen thresholds (obtained by minimizing the distance between $\hat{f}(0)$ and *f*(**0**) over the grid 0.0001 to 0.002 in steps of 0.0001) is depicted by the dotted vertical line. It can be seen that the averages of the adaptively chosen thresholds are quite close to the “optimal” thresholds which lends support to the proposed adaptive method.

## Discussion and conclusions

The EM algorithm for estimating haplotype frequencies from pooled genotype data is computationally not feasible when the number of loci and/or the pool size is large due to the combinatorial challenge of finding all possible haplotypes that are compatible with the observed pool tools. Gasbarra *et al.*[20] raised the possibility of using database information to form a list of frequently occurring haplotypes, and by restricting attention to only those haplotypes on such a list, Pirinen [21] made the EM algorithm much more viable. The success of the EM with a list method is, however, dependent on the correctness of the list used. In the absence of an external list of possible haplotypes, especially for rare alleles for which there is not a lot of database information, and to protect against using the wrong list, we look at the feasibility of using the data at hand to create an internal list of possible haplotypes to be fed into the EM algorithm. Motivated by the collapsed data method studied by Kuk *et al.*[29], we propose a CD list with amplified haplotype frequencies. This alone does not work well but with appropriate augmentation and trimming, the resulting EM-ATCDL algorithm performs very well in our simulation study. It should be pointed out that even though the ATCD list originates from the CD list which is based on collapsed data: a further reduction of pooled genotype data, the EM-ATCDL estimates themselves are computed using the pooled data, which explains why they are better than the collapsed data MLEs. The simulation results also suggest that augmenting the collapsed data list alone, or trimming the list alone, is not good enough, and it is necessary to do both. The average lengths of the various lists are also shown in Tables 3 and 4. We can see that the average length of the ATCD list ranges from 20 (*k*=1,*n*=100) to 30 (*k*=4,*n*=200) for the 25 loci case, and from 28 (*k*=1,*n*=100) to 36 (*k*=4,*n*=200) for the 32 loci case. Without using a list, there are 2^25^≈3e7 and 2^32^≈4e9 possible haplotypes. Thus by using the ATCD list, we can restrict our attention to only 20 to 40 haplotypes, hence the huge savings in running time. It can also be seen from Tables 3 and 4 that making a list longer does not guarantee better results, as the EML and CD lists are much longer than the ATCD list but the resulting estimates are much worse. What seems important is to add the right haplotypes and remove unnecessary ones. If an imperfect external list exists, then a sensible hybrid method is to combine it with the collapsed data list to form a union list which can be further augmented and trimmed using the techniques described in this paper.

Currently we are only adding haplotypes with a single “1” to the list, which seems reasonable for the study of rare variants, but one can conceivably also add haplotypes with two 1’s to the list. This will increase the number of possibilities substantially during the first iteration of the EM algorithm, but most of these haplotypes will be removed after one iteration.

The signs are promising that the use of the ATCD list can push the limit of the EM algorithm in terms of the number of loci and pool size that it can handle. This method is particularly well suited for estimating the haplotype distributions of rare variants which are of substantial current interest. Note that our method does not require sampling, and is shown in simulation study to work for case of 32 loci and pool size 4, which is beyond the scope of most sampling-based methods, MCMC or deterministic.

## Methods

### Definitions and notation

Focusing on bi-allelic loci, the two possible alleles at each locus can be represented by “1” (the minor or variant allele) and “0” (the major allele). As a result, the alleles at selected loci of a chromosome can be represented by a binary haplotype vector. Since human chromosomes come in pairs, there are 2 haplotype vectors for each individual, one maternal, and one paternal. Suppose we have *n* pools of *k* individuals each so that there are *K*=2*k* haplotypes within each pool. Denote by *Y*_*ij*_=(*Y*_1*ij*_,⋯,*Y*_*Lij*_) the *j*^*th*^ haplotype in the *i*^*th*^ pool, where *i*=1,⋯,*n*, *j*=1,⋯,*K*, and *L* is the number of loci to be genotyped. Assuming Hardy-Weinberg equilibrium, the *nK* haplotype vectors are independent and identically distributed with probability function

$$f(y_{1},\cdots \;,y_{L})=P\left(Y_{1\text{ij}}=y_{1},\cdots \;,Y_{\text{Lij}}=y_{L}\right)$$

for every *L*-tuple *y*=(*y*_1_,⋯,*y*_*L*_) belonging to the Cartesian product *Ω*={0,1}^*L*^. With pooling, the observed data are the pool totals

$$\begin{array}{l} T_{i}=\overset{K}{\underset{j=1}{\sum }}Y_{\text{ij}}=\left(\overset{K}{\underset{j=1}{\sum }}Y_{1\text{ij}},\cdots \;,\overset{K}{\underset{j=1}{\sum }}Y_{\text{Lij}}\right)\\ \;=\left(T_{1i},\cdots \;,T_{\text{Li}}\right),\;i=1,\cdots \;,\mathrm{n.} \end{array}$$

The probability function *p*(*t*_1_,⋯,*t*_*L*_) of each pool total is given by the *K*-fold convolution of the haplotype probability function *f*(*y*_1_,⋯,*y*_*L*_) and so the likelihood based on the observed pooled data is highly intractable and not easy to maximize directly.

Kuk *et al.*[29] defined the collapsed data via indicator functions as

$$\begin{array}{l} Z_{i}=\left(I\;\left\{\overset{K}{\underset{j=1}{\sum }}Y_{1\text{ij}}\geq 1\right\},\cdots \;,I\;\left\{\overset{K}{\underset{j=1}{\sum }}Y_{\text{Lij}}\geq 1\right\}\right)\\ \;=\left(Z_{1i},\cdots \;,Z_{\text{Li}}\right). \end{array}$$

Note that what *Z*_*i*_ does is to collapse each total allele frequency to either “0” (coded as 0) or “at least 1” (coded as 1) as done in classical group testing [31]. From here on, we will call {*Y*_*ij*_,*i*=1,⋯,*n*,*j*=1,⋯,*K*} the complete haplotype data (usually not observed); {*T*_*i*_,*i*=1,⋯,*n*} the pooled genotype data (reduces to individual genotype data if the pool size is 1), and {*Z*_*i*_,*i*=1,⋯,*n*} the collapsed data. In this paper, we refer to *k* as the pool size, not *K*.

### The collapsed data maximum likelihood estimator

Suppressing its dependence on the pool size *k*, let

$$g(z_{1},\cdots \;,z_{L})=P(Z_{1i}=z_{1},\cdots \;,Z_{\text{Li}}=z_{L})$$

be the probability function of the collapsed data, and

$$g_{0}(\Lambda )=P\left(Z_{\text{li}}=0,l\in \Lambda \right)$$

the probability of zero pool totals at a subset *Λ* of the *L* loci. Kuk *et al.*[29] argued that the MLE of *g*_0_(*Λ*) based on the collapsed data *Z*_1_,⋯,*Z*_*n*_ is given by

$${ĝ}_{0}(\Lambda )=\frac{n_{Z0}(\Lambda )}{n},$$

where *n*_*Z*0_(*Λ*) is the number of pools with zero allele counts at the positions specified by *Λ*.

By making use of the relationship $f_{0}(\Lambda )=g_{0}{\left(\Lambda \right)}^{\frac{1}{K}}$, where *f*_0_(*Λ*)=*P*(*Y*_*lij*_=0,*l*∈*Λ*), and the inclusion-exclusion principle, Kuk *et al.*[29] obtained

(1) $$\begin{array}{l} {\hat{f}}_{\text{CD}}(y)={\left\{\frac{n_{Z0}\left(\right)\Lambda (y)}{n}\right\}}^{\frac{1}{K}}\\ \;+\overset{m}{\underset{r=1}{\sum }}{\left(-1\right)}^{r}\;\underset{\begin{array}{c} S\subset \Lambda^{\prime }(y)\\ |S|=r \end{array}}{\sum }{\left\{\frac{n_{Z0}\left(\right)\Lambda (y)\;\cup \;S))}{n}\right\}}^{\;\frac{1}{K}} \end{array}$$

as the collapsed data MLE of *f*(*y*), where *Λ*(*y*)={*l*:*y*_*l*_=0} stores the positions of the 0’s in *y*=(*y*_1_,...,*y*_*L*_), with complement *Λ*^′^(*y*)={*l*:*y*_*l*_=1}, which stores the positions of the 1’s, and *m* is the number of 1’s in the haplotype vector *y*.

Since the collapsed data is a reduction of the pooled data, the collapsed data MLE is less efficient than the pooled data MLE. Kuk *et al.*[29] showed that the loss of estimation efficiency due to the collapsing of pooled data is not large for rare variants and small pool size. However, if the pool size is moderate or large, which is recommended from the cost saving point of view, an estimator based on the original pooled data without collapsing can be substantially more efficient than the collapsed data MLE. This is why we want to modify the EM algorithm for finding the pooled data MLE to make it computationally feasible.

### The EM algorithm based on the collapsed data list with augmentation and trimming

If the individual haplotypes *Y*_*ij*_, *i*=1,⋯,*n*, *j*=1,⋯,*K*, were actually observed, then the population haplotype distribution function can be estimated simply by the empirical haplotype distribution. In other words, the so-called complete data MLE of *f*(*y*), *y*∈*Ω*, is

(2) $${\hat{f}}_{C}(y)=\frac{m(y)}{\text{nK}},$$

where $m(y)=\overset{n}{\underset{i=1}{\sum }}\overset{K}{\underset{j=1}{\sum }}I\left(Y_{\text{ij}}=y\right)$ is the number of times *y* appears in *Y*_*ij*_. The E-step of the EM algorithm involves taking conditional expectation of *m*(*y*) given the observed data and current estimates ${\hat{f}}^{\left(t\right)}(y),\;y\in \Omega$, to get

$$\begin{array}{ll} {\hat{m}}^{\left(t\right)}(y)=E\left\{m(y)|T_{1}=t_{1},\cdots \;,T_{n}=t_{n}\right\}\;\\ \;=\overset{n}{\underset{i=1}{\sum }}\overset{K}{\underset{j=1}{\sum }}P\left(Y_{\text{ij}}=y|T_{i}=t_{i}\right)\; & \;\\ \;=\overset{n}{\underset{i=1}{\sum }}\text{KP}\left(Y_{i1}=y|T_{i}=t_{i}\right),\; & \; \end{array}$$

where

(3) $$\begin{array}{l} \;P\left(Y_{i1}=y|T_{i}=t_{i}\right)=\frac{P\left(Y_{i1}=y,T_{i}=t_{i}\right)}{P\left(T_{i}=t_{i}\right)}\\ \;=\frac{\underset{\begin{array}{c} y_{2}\in \Omega ,\cdots \;,y_{K}\in \Omega \\ y+y_{2}+\cdots +y_{K}=t_{i} \end{array}}{\sum }\left\{{\hat{f}}^{\left(t\right)}(y)\overset{K}{\underset{j=2}{\prod }}{\hat{f}}^{\left(t\right)}(y_{j})\right\}}{\underset{\begin{array}{c} y_{1}\in \Omega ,\cdots \;,y_{K}\in \Omega \\ y_{1}+\cdots +y_{K}=t_{i} \end{array}}{\sum }\left\{\overset{K}{\underset{j=1}{\prod }}{\hat{f}}^{\left(t\right)}(y_{j})\right\}} \end{array}$$

Since the complete data multinomial likelihood belongs to the exponential family, the M-step can be carried out analytically to yield the updating formula

(4) $${\hat{f}}^{\left(t+1\right)}(y)=\frac{{\hat{m}}^{\left(t\right)}(y)}{\text{nK}}$$

which is just (2) with *m*(*y*) replaced by the imputed value ${\hat{m}}^{\left(t\right)}(y)$.

The E-step of the EM algorithm is very time consuming. As one can see from (3), it involves finding all possible underlying haplotype vectors that sum up to the observed pool total. The combinatorial problem is greatly reduced if we can restrict the possible haplotypes to come from a relatively short list.

Let *R*⊂*Ω* be a reduced list of possible haplotypes obtained by whatever method. The generic EM with a list algorithm operates in the same way as the EM algorithm described above except that the updating formula (4) is only applied to *y*∈*R*⊂*Ω*, and *Ω* is replaced by *R* under the summation symbols in Equation (3).

Kuk *et al.*[29] described a combinatorial method to arrive at a reduced list *R*, but the resulting EML algorithm is still very time consuming. As can be seen from Table 2, the EML algorithm is not feasible for pool size larger than 2. Thus there is a need for alternative methods to arrive at a reduced list. Motivated by the fact that the collapsed data MLE ${\hat{f}}_{\text{CD}}(y)>0$ only for “those haplotypes *y* which coincide with at least one of the collapsed data vectors *Z*_*i*_,*i*=1,⋯,*n*, in the sample”, it seems sensible to apply the EM algorithm with haplotypes restricted to this list, which we call the CD list.Let *y* be a non-ancestral haplotype (i.e., *y*≠**0**, the vector of all zeros) with frequency *f*(*y*)>0, the probability that it is captured in a list of *n* randomly sampled haplotypes is 1−{1−*f*(*y*)}^*n*^(≈1−*e*^−*nf*(*y*)^ if *f*(*y*) is small and *n* is large), whereas the probability that it is captured by the CD list constructed from *n* pools of *k* individuals each is 1−{1−*g*(*y*)}^*n*^≈1−*e*^−*ng*(*y*)^. Thus if *g*(*y*)>*f*(*y*), the probability that *y* is captured by the CD list is higher than the probability that it is captured by direct sampling of haplotypes (not to mention the extra cost incurred in resolving the phase ambiguity to sample the haplotypes directly), and by increasing the number of pools *n*, we can make the capture probability arbitrarily large. For example, if we want the CD list to capture *y* with probability at least 1−*ε*, all we have to do is to solve 1−*e*^−*ng*(*y*)^≥1−*ε*(after Poisson approximation) to get $n\geq \frac{-\log(\varepsilon )}{g(y)}$. A sufficient condition for *g*(*y*) to be greater than *f*(*y*) is given below.

#### Lemma 1

Let *y* be non-ancestral, *g*(*y*)>*f*(*y*) if $f(0)>{\left(\frac{1}{2k}\right)}^{\frac{1}{2k-1}}$.

#### Proof

A sufficient condition for *Z*=*y* is that one of the 2*k* haplotype vectors *Y*_1_,⋯,*Y*_2*k*_ in a pool of *k* individuals is equal to *y*=(*y*_1_,⋯,*y*_*L*_), and the other 2*k*−1 haplotype vectors are all zero vectors. Thus *g*(*y*)≥2*kf*(*y*)*f*(**0**)^2*k*−1^, and the lemma follows. □

The values of ${\left(\frac{1}{2k}\right)}^{\frac{1}{2k-1}}$ for various choices of the pool size *k* are given in Table 5. Thus if the alleles are sufficiently rare in the sense that *f*(**0**) is larger than the threshold given in Table 5, then there is a better chance of capturing each non-ancestral haplotype with *f*(*y*)>0 by the CD list than by direct sampling of haplotypes. This is achieved by re-distributing the probability of the ancestral haplotype in the process of pooling and collapsing. In other words, the reason why it is possible to have *g*(*y*)>*f*(*y*) for non-ancestral *y* is because *g*(**0**)=*f*(**0**)^2*k*^<*f*(**0**). We cannot have *g*(*y*)>*f*(*y*) for all haplotypes *y* because both *g*(*y*) and *f*(*y*) must sum to 1. Table 6 shows how the probabilities are being re-distributed for a 25-loci case. The true haplotype distribution *f*(*y*) is listed in column 1 of Table 6, whereas the distributions *g*(*y*) of the collapsed data for various pool sizes are given in the subsequent columns. For non-ancestral *y*, we can see from Table 6 that *g*(*y*)>*f*(*y*), and more so when the pool size is increased (up to a point), which is good news for the CD list. For example, *f*(1,0,⋯,0)=0.0509, whereas *g*(1,0,⋯,0)=0.0839 when *k*=1, and continues to increase to 0.1143 and 0.1169 when the pool size is increased to 2 and 3. We are particularly interested in the capability of the CD list in capturing haplotypes with multiple 1’s. For the last haplotype listed in Table 6 (which contains five 1’s), *f*(*y*)=0.0034, but *g*(*y*) is 0.0097 when the pool size is 3. Thus if we have *n*=200 pools (which is one setting of our simulation study) of *k*=3 individuals each, the probability that this haplotype is captured by the CD list is 0.8577=1−(1−0.0097)^200^≈1−*e*^−200(0.0097)^=0.8563. But *g*(*y*) will also assign positive probabilities to some haplotypes *y* even though *f*(*y*)=0 since $\underset{y:f(y)>0}{\sum }g(y)<1$, which is why we propose to trim the CD list. To see how *g*(*y*) can be positive even though *f*(*y*)=0, consider the following case with just 2 loci. Suppose *f*(1,0)>0, *f*(0,1)>0, but *f*(1,1)=0. By pooling *k* individuals together, it is obviously possible to have total allele counts *T*_1_≥1,*T*_2_≥1 at both loci, and hence (*Z*_1_,*Z*_2_)=(1,1), which means that (1,1) will appear on the CD list even though *f*(1,1)=0.

**Table 5:** Sufficient conditions for non-ancestral haplotype frequencies to be increased by collapsing data

**Lower threshold of*f*(0)**				
*k*=1	*k*=2	*k*=3	*k*=4	*k*=5
0.5000	0.6300	0.6988	0.7430	0.7743

Sufficient conditions for collapsed data frequencies {*g*(*y*),*y*≠**0**} to be greater than haplotype frequencies {*f*(*y*),*y*≠**0**} for various choices of pool size *k*.

**Table 6:** Induced collapsed data frequencies

**Haplotype*y***		***f(y)***		***g(y)***			
**Positions of ‘1’s**		**TRUE**		***k=1***	***k=2***	***k=3***	***k=4***
None		0.7995		0.6392	0.4085	0.2611	0.1669
1		0.0509		0.0839	0.1143	0.1169	0.1065
2		0.0034		0.0055	0.0070	0.0067	0.0058
3		0.0436		0.0716	0.0967	0.0980	0.0883
5		0.0034		0.0055	0.0070	0.0067	0.0058
6		0.0034		0.0055	0.0070	0.0067	0.0058
9		0.0073		0.0117	0.0151	0.0146	0.0125
11		0.0034		0.0055	0.0070	0.0067	0.0058
15		0.0034		0.0055	0.0070	0.0067	0.0058
19		0.0068		0.0109	0.0141	0.0136	0.0117
20		0.0068		0.0109	0.0141	0.0136	0.0117
21		0.0034		0.0055	0.0070	0.0067	0.0058
22		0.0102		0.0164	0.0213	0.0206	0.0178
23		0.0034		0.0055	0.0070	0.0067	0.0058
24		0.0102		0.0164	0.0213	0.0206	0.0178
1, 3		0.0040		0.0117	0.0307	0.0482	0.0610
1, 9		0.0029		0.0058	0.0105	0.0135	0.0148
3, 14		0.0034		0.0057	0.0082	0.0088	0.0084
6, 7		0.0204		0.0332	0.0439	0.0435	0.0384
3, 6, 7		0.0034		0.0077	0.0164	0.0231	0.0271
1, 6, 7, 24		0.0034		0.0060	0.0097	0.0119	0.0132
1, 12, 13, 22, 25		0.0034		0.0059	0.0087	0.0097	0.0096
Sum of haplotype probabilities		1.0000		0.9751	0.8822	0.7650	0.6462

Haplotype frequencies *f*(*y*) for a 25-loci case and the induced collapsed data frequencies *g*(*y*) for various pool sizes *k*.

The CD list misses some haplotypes with *f*(*y*)>0, while some other haplotypes with *f*(*y*)=0 are erroneously included. This suggests that the CD list needs to be augmented as well as trimmed. Since we are focusing on rare variants, we augment the CD list by adding all those vectors with only one “1” to the list if they are not already there. Thus we are adding at most *L* haplotypes to the CD list. Beginning the EM iteration with the augmented CD list, we remove a haplotype from the list if its estimated frequency at the current iteration of the EM algorithm is less than a threshold. The way we select the threshold (typically over a grid) is to choose the one that results in an estimate of the ancestral haplotype frequency *f*(**0**) closest to the collapsed data MLE ${\hat{f}}_{\text{CD}}(0)$, which should be a reasonable benchmark.

## Availability

http://www.stat.nus.edu.sg/~a0068196/EM-ATCDL/

## Competing interests

The authors declare that they have no competing interests.

## Authors’ contributions

AYCK and XL contributed 40% each and JX contributed 20% to this work. All authors read and approved the final manuscript.
